# Lessons learned to improve engagement, recruitment and retention of multilingual communities: a case study of a fresh produce box program

**DOI:** 10.3389/fpubh.2025.1612491

**Published:** 2025-09-23

**Authors:** Stella S. Yi, Erinn M. Hade, Lan N. Đoàn, Sze Wan Chan, Simona C. Kwon

**Affiliations:** Department of Population Health, NYU Langone Health, New York, NY, United States

**Keywords:** community health, language, adaptation, recruitment strategies, retention strategies

## Abstract

Many communities are oftentimes labeled as “hard to reach communities” by health researchers. Instead, what may be a more appropriate framing is that conventional research approaches are a mismatch for recruiting and retaining these understudied communities. This paper describes an inclusive research process with particular attention to engaging multilingual communities in the U.S. that we have developed while implementing a community-level nutrition program. The program, Harvest Share, is an equity-centered, systems-based strategy to improve diet for neighborhood residents in Brooklyn, NY. Our research approach involves three components: participatory mapping, cultural adaptation/transcreation and language justice, and two cross-cutting areas: researcher positionality and research team diversity. The application of these methods in research resulted in our research program being highly accepted by partner organizations and participants. Applying inclusive research practices in addition to centering the community/es of interest will aid in the implementation of solutions that are feasible and culturally and linguistically responsive, ensuring successful recruitment and retention; and in the long run, sustainable, community-engaged solutions that have high acceptability and promote community well-being.

## Introduction

In the last decade, equity has emerged as a driving force in health research and more broadly in society. Yet one group that has remained invisible within health research are those who speak languages other than English. While the U.S. population has a small percentage of individuals who speak English less than very well (8%), in state and local communities, the picture shifts. In California, Texas, New York, and Florida, 18, 13, 13 and 12%, respectively, are individuals who speak English less than very well (1); in metropolitan areas, home to diverse immigrant communities, the estimate shifts to 25% (2). Nationally, Spanish is the most common language spoken by those who speak English less than very well (~60%), but this varies greatly across states, where Spanish-speakers comprise only 46% in Washington and 50% in New York amongst individuals who speak English less than very well (3). In other words, other languages (e.g., Mandarin, Hindi, Arabic, Russian, Haitian Creole) must also be considered in research approaches in addition to Spanish. The four most common languages spoken in New York City in 2024 other than English are Spanish, Chinese, Russian and Bangla (4).

Communities that speak languages other than English face socioeconomic barriers to healthcare access, contributing to overall lower health status (1, 5). Yet the majority of research continues to be conducted in English only – meaning as a group, researchers are intentionally not reaching certain communities experiencing worse health disparities. With regards to administrative data, a scan of national, state and local datasets in the U.S. (6, 7) revealed that of 39 surveys promisingly, 69% were offered in languages other than English and Spanish (unpublished data). However, the remaining 31% of surveys that only collect data in English and Spanish include major health-related databases like the Behavioral Risk Factor Surveillance Survey, the National Health Interview Survey, and the Health and Retirement Survey. Even when other languages are included, “inclusion” should be taken with a grain of salt. The National Health and Nutrition Examination Survey or NHANES, for example, is administered in English, Spanish, Chinese, Vietnamese and Korean. Yet the vast majority of NHANES participants completed the survey in English (90.8%), followed by Spanish (6.6%) (8). Only 2.6% of NHANES participants request an interpreter, and of those, 20% are Latine (8). Thus, the representation of communities that speak languages other than English is limited in health data.

Being a “hard to reach community” in research is a misnomer. What may be a more appropriate framing is that conventional research approaches, methods and strategies are a mismatch for recruiting and retaining these understudied communities – particularly those that prefer to engage in research in languages other than English. For understudied communities, mismatch may include recruitment in English only; fear of government/institutional authorities; mistrust in medical systems and the research process; and the development and implementation of interventions that do not align with community sociocultural norms/values or with community priorities – such as economic security, discrimination or employment. The success of community-partnered research initiatives lies in being attuned and adaptive toward these facets at multiple levels.

Robust yet flexible processes for successfully engaging understudied communities in research exist through community-engaged research practices. Community-partnered research practices address some challenges, such as the appropriate focus of implemented programming to address the health needs of the partnering community; mitigation of mistrust through partnerships and collaborations with a trusted community partner; and by aligning differing priorities across academic and community sectors (9). However, these methods can still fall short in reaching goals for study enrollment, especially if the timing of engagement, equanimity and transparency in financial costs of conducting research or clear communication are not carefully executed. For this paper, we will describe a three-step inclusive research process with a particular focus on communities that speak languages other than English, offering recommendations and how-to guidance for researchers to more equitably engage in community-partnered research initiatives.

## Context: a fresh product box program

Harvest Share, Our Communities, Our Foods is a program to improve diet among residents in Brooklyn, New York, that was initiated in 2021. Foundation funding supported the program from 2021–2023 and federal funding from 2022–2027 (10, 11). The program is a collaboration across 11 multi-sector partners, including academia, community organizations, farms, a food pantry, and two elementary schools, and is coordinated by an academic research team at NYU Langone Health. One of Harvest Share’s main activities is a cost-offset, community-supported agriculture (CSA) program that features Chinese and Mexican-specific fresh produce that is grown locally by two farm partners. Harvest Share is centered in Sunset Park, Brooklyn, NY—a predominantly Chinese and Mexican American neighborhood—but is inclusive of those residing in and adjacent to Sunset Park—which includes Bangladeshi American, White, and additional Latine ethnic groups—given the demonstrated interest of community members. Therefore, the Harvest Share program eligibility and enrollment criteria shifted from participants’ racial or ethnic group as initially planned and to their language preferences: English, Mandarin, Spanish and Bangla (11). Harvest Share is highly accepted by all partner organizations and participants; amongst pilot participants, we observed a 90% participant weekly pickup rate for the produce boxes for the growing season over 20 weeks in 2022 (10).

**Figure 1:**
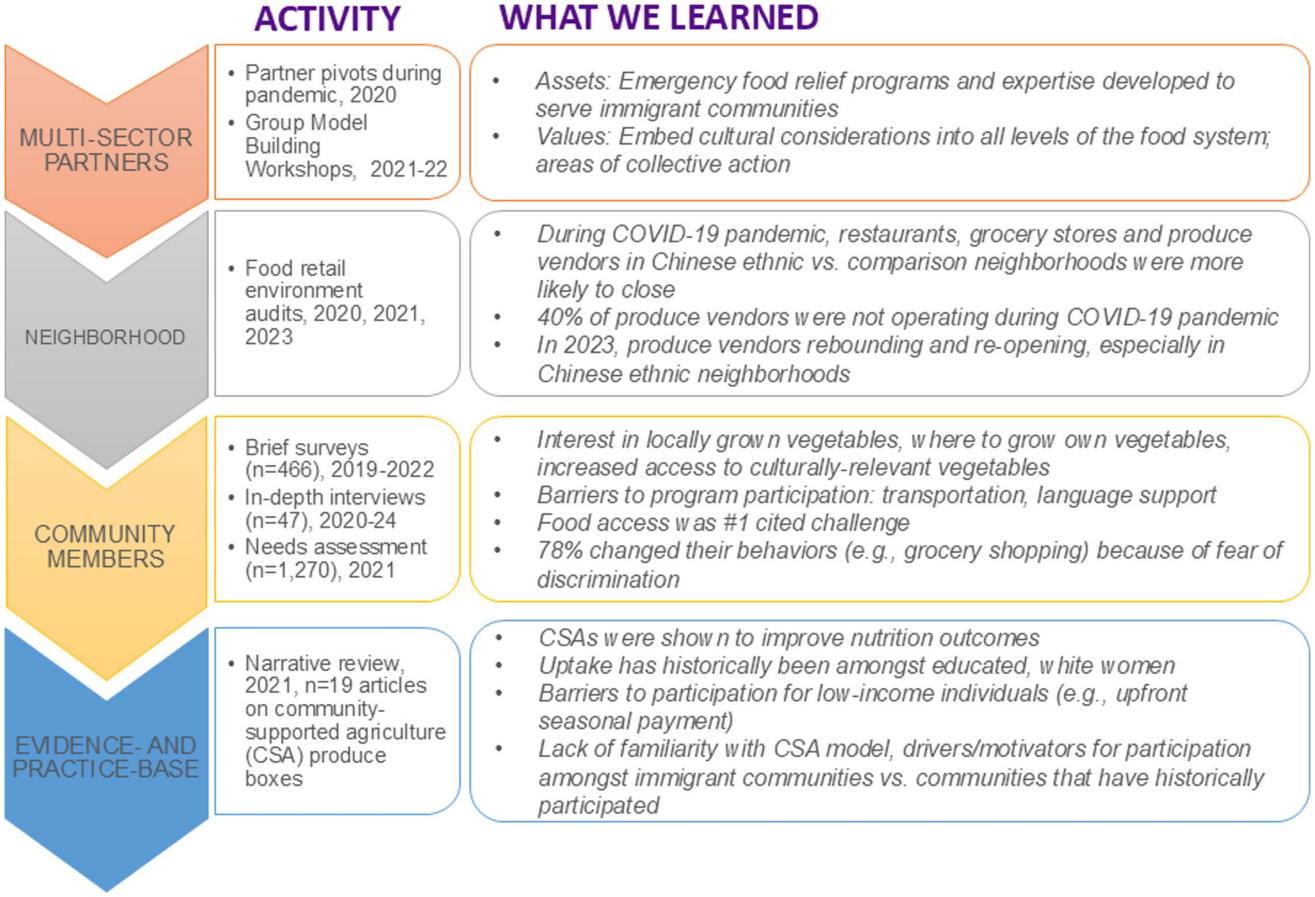
Harvest Share formative research activities.

In developing and refining the Harvest Share model we undertook a series of formative research activities from 2020 to 2024 (Figure 1). We first conducted a narrative review of *n* = 19 articles on CSA fresh produce boxes. We found that CSAs are an effective way to improve nutrition outcomes, but that uptake has historically been amongst educated, higher income and White communities in the U.S. The upfront payment model required of most CSA models has precluded participation among those with lower income. Next, we took stock of the many community partner assets that were put into place during the COVID-19 pandemic from March 2020 onwards. These assets included pivoting community-based programming to include rapid food distribution; and amongst farm partners, three activities: (1) shifting to culturally appropriate produce for charitable food efforts reflecting the needs observed in the local community; (2) gaining experience with implementing a sliding scale payment system and accepting payments weekly and via electronic benefit transfer (EBT) such as Supplemental Nutrition Assistance Program (SNAP); and (3) creating direct linkage between farms and food pantries/distribution sites. Concurrently from 2021 to 2023, we conducted brief surveys on vegetable preferences and preparation knowledge (*n* = 466), interviews on familiarity and interest in a CSA model (*n* = 48, 4 languages) (12–15), a needs assessment (*n* = 1,270) amongst community members (16); and administrative assessments of the food retail environment and availability of fresh produce (17–19). These formative activities allowed for us to learn what vegetables to grow and what seed orders were needed, and additionally demonstrated that there was an interest in locally grown vegetables, people wanted to know where to grow their own vegetables and they had an interest in interacting with farmers and other community members around fresh vegetables. Amongst the Asian American community in New York City, food access was the #1 cited challenge during the COVID-19 pandemic (16). Generally, there was little awareness of CSAs but an interest in the program model, with cited barriers being transportation and language support. Community preferences and values gleaned from these activities (e.g., family-based activities, desire to interact with farmers) were incorporated into the development of the program messaging and approach, and materials.

Lastly, we undertook group model building—a participatory systems science technique—to understand the priorities of local, multi-level and multi-sector partners and opportunities to improve diet amongst immigrant communities. Across 14 partners and 5 workshops, we identified a shared priority of embedding cultural considerations into all levels of the food system which would require coordinated action across all multi-sector partners (20). Four areas of collective action were identified: local fresh food access, nutrition education, experiential learning (e.g., gardening, cooking), and food policy change (20). These four areas plus economic security, an important driver of food access became the foundational structure for Harvest Share. As Harvest Share implementation continues, we iteratively incorporate feedback from a Chinese-speaking advisory board (*n* = 8, Mandarin, Cantonese), a Spanish-speaking advisory board (*n* = 6), and through direct interaction with participants and partners. Harvest Share also resulted in the introduction of a rooftop garden, composting workshops, hydroponics systems and culturally appropriate seedling distribution programs in local community spaces, which evolved when we learned about participant/community interest and deep knowledge of Harvest Share participants and community members towards farming/gardening as a way to maintain connection to their heritage.

Following this broad array of formative research activities, we sought to outline the values and norms of our communities of interest to apply knowledge gained to our cultural adaptation/transcreation process. Transcreation is the process of adapting a message/text from one language to another while maintaining its intent, style and tone rather than a word-for-word translation (e.g., idioms) (21). In the development of our program, marketing, messaging and program resources, we applied a transcreation lens (see example, Table 1) and incorporated both surface and deep cultural adaptations. For example, surface adaptations included utilizing Chinese and Mexican produce and recipes, while deep adaptations included modifying offered bunch sizes of produce to align with cultural recipes. We also learned from the interviews described above that community members wanted access to culturally appropriate fresh produce, and needed in-language nutrition resources. We learned that Chinese and Mexican American immigrant participants were more likely to prioritize freshness of produce over price and proximal stores (22, 23). We worked with a social marketing agency to create a cohesive brand and look for materials across all languages. Participant fliers for the Chinese American community were developed in simplified Chinese then back-translated into English; and materials were developed to appeal a wide range of community members not typically considered (e.g., English-speaking racial and ethnic groups).

**Table 1:** Harvest share program name translation/transcreation and considerations.

Language	Name and tagline	Translated meaning	Translation and context considerations
English	Harvest Share Our Communities, Our Foods	–	Double meaning of “share” – something enjoyed with others and a farm share
Simplified Chinese	共享丰收 我们的社区 我们的食材	Sharing the Harvest/Enjoying Harvest Together Our Community Our Foods	Double meaning of “共享” – sharing and enjoying together
Spanish	Cosecha Colectiva Nuestras Comunidades, Nuestros Alimentos	Collective Harvest Our Communities Our Foods	Double meaning of “share” does not translate well, so pivoted to focus on ideas of collectiveness and harvest
Bangla	উৎপাদিত সবজি বিতরণ আমাদের সমাজ, আমাদের খাদ্য	Distribution of Crops/Harvest Our Communities and Our Foods	Whether to use Bangla script or romanized Bangla when translating

Honoring local community preferences, we employ live interpretation and written translation/transcreation which is implemented by our diverse bicultural bilingual research team and partners. Live interpretation includes bilingual staff support at in-person events (e.g., cooking workshops, gardening events, farm tours) as well as during bimonthly virtual and in-person Harvest Share partner meetings. One Harvest Share partner feels more comfortable speaking in Spanish. Therefore, we offer live interpretation on every virtual call and in-person meeting this partner participates in and have the expectation that all Harvest Share partners in attendance actively practice language justice principles (e.g., translating PowerPoint slides/chat messages to Spanish, speaking slowly to allow for accurate, live interpretation). Translations of participant materials have been managed by both target language message development and back translation and by multiple native language speakers’ translation from English and review. In addition to ensuring translation accuracy, the review process also serves as a check that context of translated language is culturally congruent.

In implementing Harvest Share, we additionally consider and revisit our positionality as researchers. Questions such as, “In what ways have you reflected on your identity and positionality when conducting this research?” and “Do you have the capacity to engage and serve the community appropriately?” have served as an important starting point for individual contemplation and group discussion. In this vein, we give equal weight to all partner and community members’ ideas. Harvest Share participants have offered their professional services and have become paid consultants on our project (e.g., arts sessions for kids). Harvest Share partner-led ideas have been incorporated into workshop content and program flow; as a result of partner suggestions, we implement an annual block party, farm tours and field trips; and conducted an availability/price audit of produce offered in the CSA boxes compared to local neighborhood stores (45). We also actively support partners on their own grantmaking initiatives and invite partners to be co-authors on published manuscripts, sponsor their conference attendance and co-present on the Harvest Share program. Owing to the skills and identities of our research team for each target language in Harvest Share we have or engage with: an equity-focused faculty researcher with deep trust/commitment to and/or from the community of interest; a bilingual project coordinator; bilingual undergraduate or graduate-level student interns; community-based organization that directly serve the community of interest; and community members themselves. Lastly, since the start of Harvest Share we have been co-developing a multi-pronged sustainability strategy with partners for program activities (24).

## Three-step process for more inclusive research

**Figure 2:**
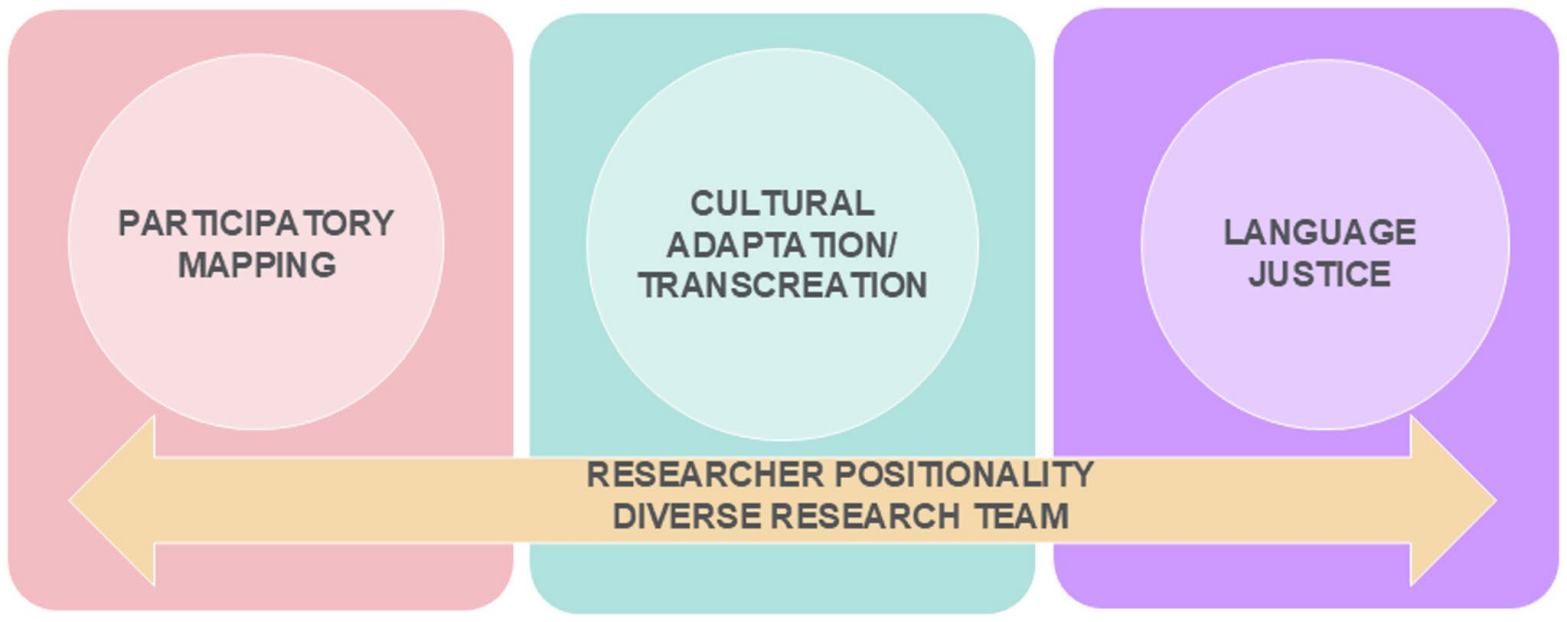
A 3-step process for more inclusive research drives Harvest Share implementation.

We have simultaneously developed and applied a three-step process for more inclusive research in the implementation of Harvest Share (Figure 2). Our approach additionally draws from our experience implementing community-engaged research projects since 2003, partnering with communities who have been historically underrepresented in research; a non-exhaustive but exemplary list includes those who speak Mandarin, Cantonese, Korean, Bangla, Tagalog, Japanese, Spanish, Arabic, Haitian Creole and Russian (10, 16, 25–36). Each of the components of the inclusive research process is described in detail below.

### Step 1: participatory mapping

The first component is the participatory mapping step. Participatory mapping refers to a “mapping” of community member and partner needs, priorities and assets with practice-and evidence-based approaches in the published literature. One can start by outlining an understanding of the values, cultural norms, and health needs of the local community of interest and the partners who serve them, using mixed methods. This knowledge gathering process may take the form of more formalized research procedures such as quantitative surveys, focus groups/interviews or examination of local administrative data; or more informal approaches such as listening sessions or brief feedback surveys. Researchers may then conduct a comprehensive appraisal of the existing published literature of evidence-based interventions that have been demonstrated to address the community outcome of interest. In some cases, the intervention may be scaled to similar populations as those in which it was developed and tested, while in others, the programs are culturally modified and adapted for previously unreached populations. In the latter scenario, challenges can arise if the intervention is something the community of interest does not want, or is not well-suited to a community – not just because of language, but owing to other drivers of health, such as culture or means. The intervention should then be selected that aligns with community belief systems and priorities and available resources. As we have observed, it’s common for researchers to decide on the ‘best’ approach for a community based on effectiveness alone, introduce it into a community, and then fail to understand why the community is not receptive and the project had low engagement or retention. This mapping process should therefore be driven by community and partner input throughout development and implementation of the research. The end goal of the participatory mapping process is to have a selected practice-or evidence-based approach that is acceptable to the community, the community partners and guided by the most up-to-date science.

### Step 2: cultural adaptation/transcreation

In tandem with the participatory mapping process is the cultural adaptation/transcreation process. The cultural adaptation/transcreation process is an in-depth examination of the recruitment, engagement and retention approaches for participants, including materials, wording, imagery and language; as well as consideration of other aspects of program design, evaluation approach, outcomes measures or program content that may be modified by community preferences or culture. The cultural adaptation/transcreation should be viewed as an iterative observation and documentation across all the formative sources, evidence base and local community assets to create culturally appropriate programs, resources and materials.

### Step 3: apply a language justice lens

Language justice is “an evolving framework based on the notion of respecting every individual’s fundamental language rights—to be able to communicate, understand and be understood in the language in which they prefer and feel most articulate and powerful” (37). Broadly, language access is making materials available in different languages. A language justice approach fosters bi-directional communication, allowing for active participation from individuals who speak different languages (37, 38).

From the outset, the researcher’s goal is to reach all individuals in the priority population, rather than introducing research in English only. If your budget is constrained, consider conducting your study in a language other than English and practice a transcreation approach in adapting and translating materials. Key considerations for implementing a language justice approach include understanding the spoken and written language needs the community has; building in adequate time and funding for multiple language translations; and identifying individuals and/or organizations that may assist in the translation/transcreation process as equitably compensated consultants. Examples of language justice components that can be employed include: (1) providing live interpretation; (2) translation of participant materials; and (3) hiring diverse, bilingual staff. Live interpretation may happen at in-person events or on virtual meeting calls. For participant-facing recruitment materials, research teams may opt to develop social marketing messages in the target language first, then back-translate into English for quality and content assurance. This creates a cohesive and appropriate linguistic style that is more attractive to participants speaking that target language. For other study documents including surveys, consent forms and interview guides (if applicable), researchers may employ a multi-step translation process. We have determined the ideal process for translations of materials into multiple languages is for the material to be independently translated by two native speakers, then reviewed by a third native speaker. There are modifications to this process, whereby only two native speakers are available; or if multiple language translations are required in a shorter time frame.

### Cross-cutting: researcher positionality and diverse research team

Researcher positionality refers to the researcher’s own background, social identities, and experiences, which shape their worldview and influence their research. A diverse research team is one that includes individuals with a variety of backgrounds, experiences, and perspectives. This may include demographic factors like race, ethnicity, gender, and age, as well as non-demographic factors such as different areas of expertise, professional experience, and thinking styles. A critical lynchpin for the success of community-engaged projects lies in examining one’s positionality as a researcher and hiring a bilingual and diverse team. This ensures that linguistic and cultural preferences are imbued throughout the research process. The team engages iteratively throughout the research process, providing feedback on the intention, approach, alignment with local norms, question wording and evaluation acceptability, continued community engagement and communication and reporting/dissemination and importantly, for the research team lead to listen, be flexible and incorporate feedback meaningfully. Ideal teams understand the languages and cultures of your community and do not consist of one token team member, but a multitude of individuals across leadership, staff, and partners. Ask yourself: how will your research team foster trust and rapport building throughout the research project and beyond?

## Discussion

We summarize challenges that may be faced by researchers and propose accompanying solutions for each challenge to improve inclusive research participant recruitment, engagement and retention of communities that speak languages other than English. These challenges include intervention or research mismatch to the community’s needs, priorities or values; a lack of diversity on a research team; and a program that only reaches the English-speaking community (Table 2). While our examples draw from working with communities that speak languages other than English, the practices we recommend apply in some part to working in partnership with other marginalized communities.

**Table 2:** Challenges and lessons learned to improve research participant recruitment, engagement and retention.

Challenge	Description/Example	Lessons learned	Action steps
Intervention/research mismatch to community needs, priorities or values	Intervention/research design may not be suited to the community belief systems or values,	Know your priority community What community are you trying to reach? What are their belief systems around the specific health topic you are seeking to understand? Select, shape & communicate on intervention/design, e.g., health research rationale, understanding biospecimen acceptability	Participatory mapping Review of evidence-and practice-base for health topic of interest Mixed methods research Systems science informed methods (e.g., group model building) Cultural adaptation/transcreation Establish cultural adaptation goals Utilize transcreation to build in deep and surface cultural relevance of research Member-checking with community partners
Research team lacks diversity	Limited representation of bicultural, bilingual perspectives on research team	Build your team mindfully Do you have the capacity to serve the community appropriately? Who do you need to add? Prioritize bilingual, bicultural staff, support and partners	Collaborate with diverse partners across academia, community and government Prioritize local community knowledge and/or bilingual skills – reflective of languages spoken in your community of interest
Program only reaches English-speaking community	Linguistic translations are of poor quality, e.g., literal translations of idioms such as, “the ball is in your court”	Do more than English What languages does the community speak? Seek to reach whom is present, rather than only those who speak English Employ a multi-step translation process with native speakers	Consider multiple facets of language justice within programming and budget: Live interpretation (in-person, virtual meetings) Materials translation Bilingual staff/partners

The success of participatory mapping and cultural adaptation/transcreation processes are dependent on existing practice-and evidence-based research. As such, it bears a reminder to consider the base of practice-and evidence-based research and our collective extant knowledge on tested and effective approaches. Who was included in pilot studies and randomized clinical trials? In what language(s) were research methods implemented in? And alluding back to positionality, who formulated the initial research protocols, approaches, and the conceptual framework of socioecological constructs that are anticipated to lead to behavior change? Not examining these core questions can eventually lead to intervention mismatch, lower engagement, and poor sustainability.

While linguistic translations and culturally appropriate imagery in health research (i.e., surface culture) have gained traction, some researchers may be only interested in ‘checking a box’ of having included such a component. Though cultural adaptation is increasingly common and recognized as being important for intervention success, many existing adaptations only focus on surface adaptations (e.g., research materials that feature a Chinese American family or Mexican cultural produce). Moreover, as a research community, the gold standard framework for health promotion interventions should be transcreation—prioritizing preservation of style, intent and tone—and not literal translation or just adaptation (39). Linguistic translations may be processed by artificial intelligence and translation companies but may have questionable quality (e.g., turkey breast recipe translated to Mandarin is human breast) and research studies might only have translated written materials but little or no budget allocated towards bicultural bilingual staff to support participant involvement in research study continuum. Translated research materials may be the first contact that a participant has with the research space, and poor-quality materials could dampen the likelihood of interest and engagement. Language justice must not stop at written translations – there must also be considerations for populations with low literacy, low health literacy and their preference for spoken language. Researchers must examine each and every communication touchpoint with participants to ensure a space that reflects language justice.

Recognition of positionality in health equity research has increased – the role it plays for the researcher’s status, perspective, value systems and decision-making processes and its influence and bias it has on research (29, 30). If research teams were more diverse and more specifically, linguistically and culturally matched the community one is trying to recruit, the resulting research, approaches, and troubleshooting would more appropriately reflect the values and linguistic nuances of the community of interest, and therefore lead to better engagement, sustainability and health impact for the community. We have long employed this philosophy in our research projects, engaging community-based organization partners as a part of the research team, hiring bilingual, lay community members to serve as research liaisons, and inviting community members to be on advisory boards, leading to high engagement and retention in communities that are often labeled as “hard to reach” (30, 40–44). In recent electronic health record-based recruitment, we recruited Chinese American immigrants with gastric cancer at safety net hospitals and family-based clinics with a 47% recruitment rate of those eligible, and 85% retention over a 6-month intervention (30). We credit the success with recruitment and retention in part, to a team that includes support for each community from research faculty, staff, community partners and community members having driven the protocol development and implementation.

## Conclusion

By shifting the onus away from participants and what they are doing wrong, we must collectively reflect on what we as a research community are perpetuating and work to shift the paradigm and to engage in inclusive research practices. The foundation of an inclusive research process is a centering of research in the community of interest, which will aid in the development of solutions that are locally, culturally and linguistically responsive. We encourage public health researchers to think outside the traditional toolbox of methods and evidence, and to be open to new ways of designing and developing programs, reaching and working with communities, and adopting perhaps less rigidly academic, but feasible practice-based solutions. In this way, our research will collectively put equity in practice and more importantly, increase sustainability and community capacity towards promotion of community well-being in the long run, as well as investing and successfully establishing a diverse public health workforce.

## Data Availability

The original contributions presented in the study are included in the article/supplementary material, further inquiries can be directed to the corresponding author.
